# Fundamental Parameters Line Profile Fitting in Laboratory Diffractometers

**DOI:** 10.6028/jres.109.002

**Published:** 2004-02-01

**Authors:** R. W. Cheary, A. A. Coelho, J. P. Cline

**Affiliations:** University of Technology Sydney, Broadway, Sydney, NSW, Australia 2007; Bruker-AXS, Östliche Rheinbrückenstraβe 50, D-76187 Karlsruhe, Germany; National Institute of Standards and Technology, Gaithersburg, MD 20899-8523

**Keywords:** fundamental parameters, microstructure analysis, parafocusing optics, profile convolution, profile fitting, x-ray powder diffraction

## Abstract

The fundamental parameters approach to line profile fitting uses physically based models to generate the line profile shapes. Fundamental parameters profile fitting (FPPF) has been used to synthesize and fit data from both parallel beam and divergent beam diffractometers. The refined parameters are determined by the diffractometer configuration. In a divergent beam diffractometer these include the angular aperture of the divergence slit, the width and axial length of the receiving slit, the angular apertures of the axial Soller slits, the length and projected width of the x-ray source, the absorption coefficient and axial length of the sample. In a parallel beam system the principal parameters are the angular aperture of the equatorial analyser/Soller slits and the angular apertures of the axial Soller slits. The presence of a monochromator in the beam path is normally accommodated by modifying the wavelength spectrum and/or by changing one or more of the axial divergence parameters. Flat analyzer crystals have been incorporated into FPPF as a Lorentzian shaped angular acceptance function. One of the intrinsic benefits of the fundamental parameters approach is its adaptability any laboratory diffractometer. Good fits can normally be obtained over the whole 20 range without refinement using the known properties of the diffractometer, such as the slit sizes and diffractometer radius, and emission profile.

## 1. Introduction

The fundamental parameters approach to line profile fitting uses physically based models to generate the line profile shapes. The instrument profile shape *K*(2*θ*) is first synthesised by convoluting together the geometrical instrument function *J*(2*θ*) with the wavelength profile *W*(2*θ*) at the Bragg angle 2*θ*_B_ of the peak,

K(2θ)=∫W(2θ−2φ)J(2φ)d2φ=W(2θ)⊗J(2θ) (1)

where the function *J*(2*θ*) itself is a convolution of the various instrument aberration functions associated with the diffractometer, ie.,

$$\text{ie}.,\;J\left(2\theta \right)=J_{1}\left(2\theta \right)⊗J_{2}\left(2\theta \right)⊗\ldots ⊗J_{i}\left(2\theta \right)\ldots ..⊗J_{N}\left(2\theta \right).$$ (2)

**Fig. 1:**
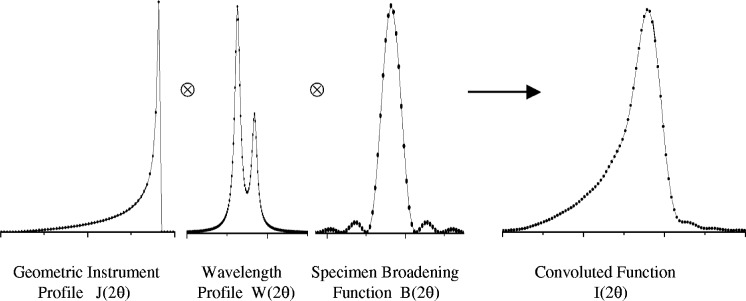
Convolution of the geometric instrument profile *J*(2*θ*), the wavelength profile *W*(2*θ*) and the specimen broadening function *B*(2*θ*) to produce the measued convoluted function *I*(2*θ*).

Diffraction broadening is incorporated into the profile function *I*(2*θ*) by convoluting the broadening function *B*(2*θ*) into the instrument profile function as shown Fig. 1,

I(2θ)=K(2θ)⊗B(2θ). (3)

This technique of profile synthesis was first introduced 50 years ago by Alexander [1], but has only been implemented as a standard fitting procedure during the last ten years [2,3,4]. More recently, freeware and commercial software packages [5,6,7] have become available for fundamental parameters profile fitting (FPPF) either for use as single line profile fitting, lattice parameter refinement or for Rietveld analysis.

FPPF has been used to synthesise and fit data from both parallel beam and divergent beam diffractometers. The refined parameters are determined by the diffractometer configuration. In a divergent beam diffractometer these include the angular aperture of the divergence slit, the width and axial length of the receiving slit, the angular apertures of the axial Soller slits, the length and projected width of the x-ray source, the absorption coefficient and axial length of the sample. In a parallel beam system the principal parameters are the angular aperture of the equatorial analyser/Soller slits and the angular apertures of the axial Soller slits. The presence of a monochromator in the beam path is normally accommodated by modifying the wavelength spectrum and/or by changing one or more of the axial divergence parameters. Flat analyser crystals have been incorporated into FPPF as a Lorentzian shaped angular acceptance function.

One of the intrinsic benefits of the fundamental parameters approach is its adaptability to any laboratory diffractometer. Good fits can normally be obtained over the whole 2*θ* range without refinement using the known properties of the diffractometer, such as the slit sizes and diffractometer radius, and the emission profile. Fine tuning is sometimes necessary to accommodate a monochromator or to compensate for the fact that certain aberrations are not completely independent [8]. Under these conditions some of the instrument parameters need to be refined, but the refined values normally are within ±10 % of the actual values. Correlation between refined instrument parameters can occur when fitting to data over a restricted 2*θ* range. Such correlation occurs between the axial divergence parameters and absorption as both of these aberrations can produce similar forms of asymmetric profiles between 2*θ* = 50° and 100° in diverging beam diffractometers. Correlation is minimised by using data with a large 2*θ* range so that the unique angular dependence of individual aberrations becomes evident. When a set of instrument profiles cannot be fitted by FPPF, this is usually an indication that either the model used is invalid (eg. incorrectly chosen slit value), the instrument is misaligned, there is an overlapping impurity line or, the specimen is generating crystallite size broadening or is inhomogeneously strained.

FPPF was designed originally as a tool for analysing diffraction line broadening. Fitting is done by convolution and corrections for instrument broadening and peak shift are intrinsic to the refinement. When an instrument is well characterised, line broadening can be analysed without a reference specimen. Moreover, when a reference standard is used, which has different properties from the specimen with line broadening, some compensation can be made for these differences. For example, when LaB_6_ SRM 660a (*µ*_powder_ ≈ 500 cm^−1^) is used as a reference, compensation can be made for differences in the absorption of the sample and LaB_6_. In the latest version of the commercial software package (TOPAS)^2^, the concept of fundamental parameters has been extended so that any user defined profile that accurately describes the physical broadening can be readily convoluted into the refinement.

In this paper we will discuss the physical origin of the instrumental profile shapes for various laboratory diffractometer configurations including both divergent beam and parallel beam instruments. This will include a description of the geometrical aberrations as well as discussion on the nature of the wavelength distribution and the influence of monochromators on this distribution. Some discussion is also presented to demonstrate how the FPPF may be fitted to experimental data from a material with a low attenuation coefficient.

## 2. Basic Objectives of the FPPF Technique

**Fig. 2:**
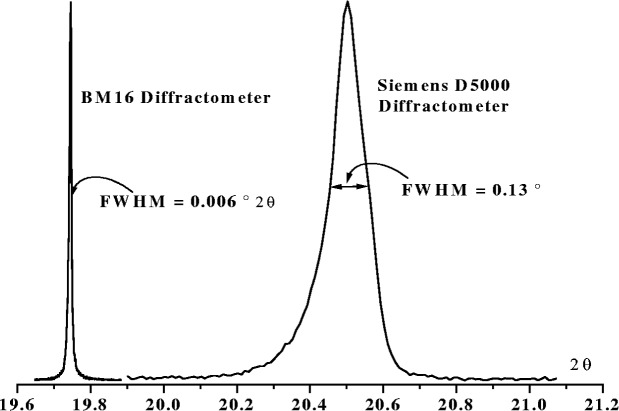
Comparison of profiles produced by the reference specimen LaB_6_ (SRM 660a) at similar 2*θ* angles on two different diffractometers, (a) on the high resolution powder diffractometer BM16 at the ESRF, Grenoble (*λ* = 0.35 Å) and, (b) on a conventional *θ*-2*θ* divergent beam diffractometer (*λ* = 1.541 Å) with a radius of 217 mm and commonly used slit sizes (ie. 1° divergence slit, 0.2 mm receiving slit and 2° Soller slits in the incident beam).

One of the basic objectives of the FPPF technique is to be able to fit any powder diffraction profile using a physically based model to describe both the instrument profile and any diffraction broadening generated by the specimen. In principle, therefore, the technique should be adaptable to any powder diffractometer and fit profiles of widely differing shapes, such as those in Fig. 2, by simply modifying the physical parameters of the diffractometer used to describe the profile.

Although most applications of FPPF have focussed on the conventional diffractometer it has also been utilised for analysing neutron diffraction data [9] and synchrotron data [10,11]. In the TOPAS implementation of FPPF there are a wide variety of possible aberration functions available within the package and these can put together to suit a particular diffractometer design and in terms of parameters that are relevant to the instrument. One of the most important achievements of the FPPF technique for practical users is speed of calculation. Accurate multiple convolution calculations over large 2*θ* ranges can be very time intensive and it is of central importance to minimise this time to enable Rietveld refinement to be completed in “seconds” without loss of accuracy within the profile function synthesis procedures. Various procedures have been implemented, some of which are described by Cheary and Coelho [2,3,4], but one of the most important has been to code the time intensive calculations at an assembler code level taking steps to optimise the use of the various registers within the PC chip.

## 3. Laboratory Diffractometer Configurations and Their Geometrical Aberrations

**Fig. 3:**
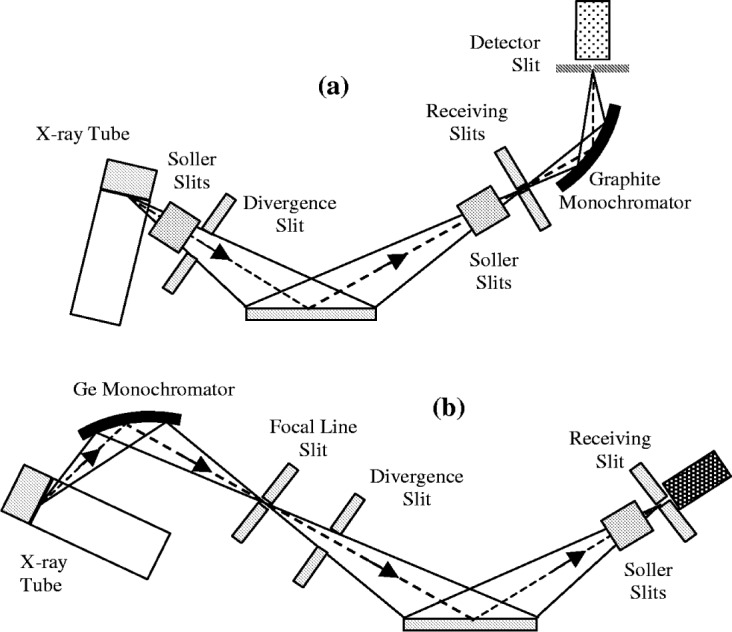
Two configurations of diverging beam diffractometer showing the principal optical components, (a) with a diffracted beam monochromator and, (b) with an incident beam monochromator.

Up until the mid-1990s most of the laboratory powder diffractometers in use were divergent beam instruments with a narrow receiving slit, diffracted beam monochromator and a simple proportional/scintillation counter detector as shown in Fig. 3a. Over the past 10 years the number of diffractometer options available from manufacturers has increased and users operate with a wider range of x-ray optical designs. The types of geometrical aberrations encountered is somewhat broader than those discussed in the classic work by Wilson [12] as will be discussed below.

### 3.1 Divergent Beam Diffractometers—Symmetric Diffraction

The most widely used laboratory diffractometer in use today is still the divergent beam diffractometer with either a bent graphite monochromator in the diffracted beam (see Fig. 3a) or, a ground and bent asymmetrically cut germanium monochromator in the incident beam (see Fig. 3b). Both of these configurations possess a similar array of geometrical aberrations. The major difference between them is the wavelength distribution which normally consists of both the K*α*_1_ and K*α*_2_ components of the K spectrum in the graphite monochromator case and only the K*α*_1_ with the Ge monochromator [13]. Further discussion of the wavelength distribution and the effects of monochromators is given later.

The principal geometric aberrations contributing to profiles from the above diffractometers are,

- the finite width of the x-ray source,
- a divergent incident beam on to a flat specimen (flat specimen error),
- the finite width of the receiving slit,
- the beam penetration into the specimen (specimen transparency),
- the deviation of the beam from the equatorial plane (axial divergence).

These aberrations all produce some degree of line broadening and, in the case of flat specimen error, specimen transparency and axial divergence, some asymmetry is also introduced. Zero 2*θ* and specimen surface displacement errors may also be present in a diffractometer, but these only affect the 2*θ* position of a profile and not its shape. In both configurations the monochromators not only determine the wavelength distribution, but they also act to reduce the axial divergence and it is often considered unnecessary to include Soller slits between the sample and the monochromator.

### 3.2 Divergent Beam Diffractometers—Asymmetric Diffraction

**Fig. 4:**
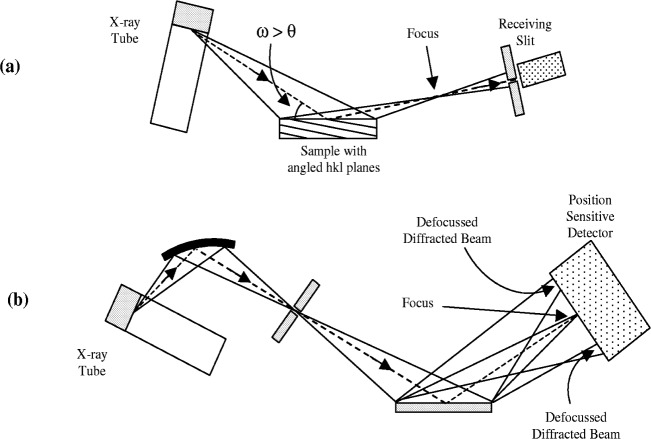
(a) Diverging beam diffractometer operated in asymmetric mode with defocussed diffracted beam at the receiving slit, (b) diffractometer with PSD replacing the receiving slit. Diffracted beam is in focus at the centre of the detector and defocussed in off-centre positions.

Divergent beam diffractometers used under symmetric conditions only measure diffraction from planes parallel to the specimen surface. To measure diffraction from planes angled relative to the specimen surface it is necessary to operate under asymmetric conditions as illustrated in Fig. 4a. The problem with operating in this mode on most commercial diffractometers is that the receiving slit is no longer at the focus of the diffracted beam and profiles are broadened by “defocussing”. The amount of defocussing is determined by the angle of divergence of the incident beam and distance of the focus from the receiving slit.

Defocussing also occurs in diffractometer configurations where,

- the sample is oscillated ±δ*ω* about the diffractometer axis so that the angle of incidence on to the specimen varies between *θ* + δ*ω* and *θ* − δ*ω*. This oscillation moves the focus of the diffracted beam continuously back and forth in front of and behind the receiving slit,
- the receiving slit is replaced by a position sensitive detector (PSD). The only position on the detector that is normally in focus is its centre (see Fig. 4b); all diffracted beams entering the detector at off-centre positions are defocussed. In PSD systems, the aberrations contributing to a profile include all the standard diffractometer aberrations, except the aberrations formerly due to the receiving slit are replaced by defocussing, the discharge resolution of the detector, and parallax error [14,15]. In a scanning PSD diffractometer, the recorded profile shape is an average of all the profile shapes across the active window length.

### 3.3 Parallel Beam Diffractometers

**Fig. 5:**
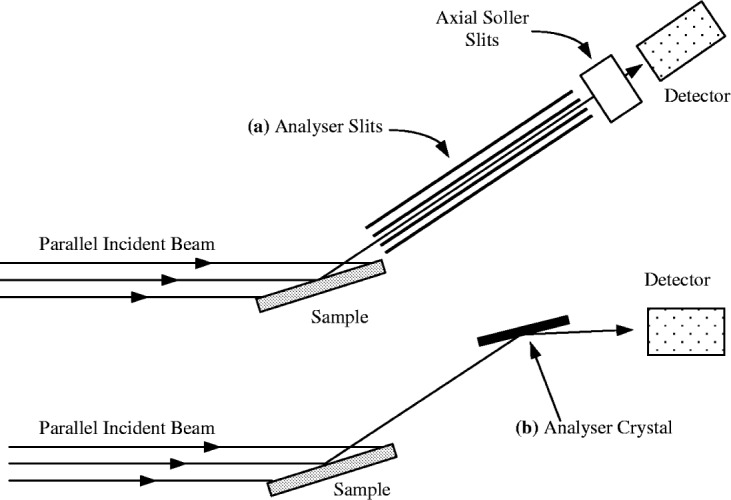
Two configurations of parallel beam diffractometer, (a) using analyser slits in the diffracted beam and, (b) using a flat analyser crystal.

There are two common forms of the parallel beam powder diffractometer which are illustrated in Fig. 5. These are based on using either analyser slits (otherwise referred to as equatorial Soller slits) or a flat Ge/Si analyser crystal as the angular discriminator of the diffracted beam. Amongst laboratory diffractometers the analyser slit set-up is the most widely used form as it offers more intensity but poorer resolution than the analyser crystal set-up.

Parallel beam diffractometers have emerged as one of the most popular forms of laboratory diffractometer over the past ten years and now constitute more than 30 % of the new diffractometer purchases. There are fewer geometric aberrations contributing to the profile and systematic errors arising from specimen displacement, specimen transparency and surface roughness are not significant. There are two geometric aberrations contributing to a parallel diffractometer,

- the angular acceptance function of the analyser foils or analysing crystal,
- deviation of the beam from the equatorial plane (ie. axial divergence).

In most laboratory diffractometers, the parallel beam is produced by using a parabolic graded multilayer mirror with the line x-ray source positioned at the focus of the mirror [16]. Although the beam may be parallel in the equatorial plane, it will not be parallel in axial plane and axial divergence can be expected in both the incident and diffracted beams. Low angle profiles will therefore be asymmetric although not to the same extent as diverging beam instruments.

## 4. The Instrument Aberrations

The geometric instrument aberrations tend to determine the shape of a diffractometer profile at low 2*θ* angles (ie., 2*θ* < 50°). At high 2*θ* angles (2*θ* > 100°), the profile conforms primarily to the shape of the wavelength distribution in the beam. With the exception of the aberrations associated with “receiving system” of the diffractometer and the x-ray source, all of the geometric instrument aberration profiles vary with 2*θ*. In the following sections the shapes of the major instrument aberrations used in FPPF analysis to describe the various laboratory diffractometer configurations are discussed for conditions that are typical of those encountered in practice. The aberration functions generated by mis-setting a diverging beam diffractometer or using it under asymmetric conditions are also discussed. Most of the results quoted here are for a diffractometer radius *R* = 215 mm.

The convention adopted here for describing the angular variables is that 2*ϕ* refers to the continuously variable angle measured on the diffractometer whereas 2*θ* or 2*θ*_B_ refers to the Bragg angle of the diffraction line. The angle *ε* refers to the difference between the measured angle 2*ϕ* and 2*θ*,

ε=2ϕ–2θ. (4)

### 4.1 Finite X-Ray Source Width

**Fig. 6:**
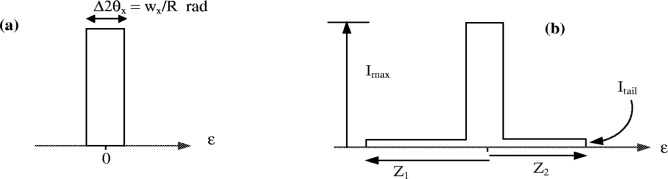
Aberration profile models, (a) simple model for a source of projected width *w_x_* and a diffractometer radius *R*, (b) model with “tube tails” containing additional parameters *f* = *I*_tail_/*I*_max_ and angular widths *Z*_1_ and *Z*_2_ from the central maximum.

The profile shape of this aberration is generally expressed as an impulse function of width Δ2*θ_x_* as shown Fig. 6a. Although the choice of an impulse function may be not be strictly valid for describing the x-ray source aberration, the exact shape used is not critical when a long fine focus tube (target width ≈0.4 mm) is installed on the diffractometer. At a take-off angle of 6° this appears as a projected width *w_x_* ≈ 0.04 mm and the aberration profile has a width Δ2*θ_x_* ≈ 0.01° and does not contribute significantly to the overall width of the instrument profile.

In broad focus tubes, the target width ≈2 mm and *w_x_* ≈ 0.2 mm at 6° take-off so that the aberration profile width Δ2*θ_x_* ≈ 0.056°. At this level the source width makes a much bigger contribution to the overall width of the instrument profile and a more accurate form for the aberration profile shape is necessary. A good approximation under these circumstances is a Gaussian shape rather than an impulse function. In diffractometers with curved crystal incident beam monochromators the source width can also have a greater contribution because of the magnification effect introduced by the monochromator. This occurs with asymmetrically-cut Johansson incident beam monochromators where the source-crystal and crystal-focal point distances are typically ≈120 mm and ≈230 mm, respectively. A fine focus tube with a projected width of 0.04 mm is then effectively magnified to ≈0.08 mm. Under these conditions the effective source width can be trimmed down by reducing the width of the focal line slit.

**Fig. 7:**
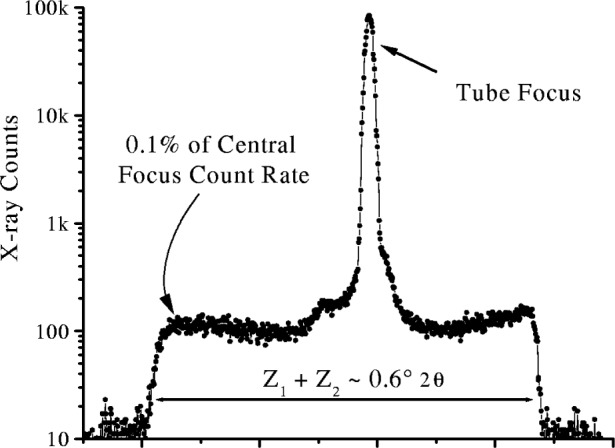
Intensity scan with 50 µm wide slit of an image formed through a 10 µm pinhole in platinum of the 0.4 mm wide long fine focus in a Cu anode x-ray tube set at 40 kV, 40 mA.

For accurate line profile analysis it is also necessary to modify the simple impulse model even with long fine focus tubes. Bergmann [17] has shown that most of the anode surface in an x-ray tube produces x rays albeit at a much lower intensity than the focal line on the anode. This is illustrated in Fig. 7 which shows the intensity recorded by scanning with a 50 µm slit across the image of a x-ray source formed through a 10 µm pinhole in platinum. A better approximation to the aberration function is a sharp impulse function superimposed on a broad impulse function to represent the so called “tube tails”. This is illustrated in Fig. 6b. The parameters introduced to describe the “tube tails” are the extents of the high and low angle tails, *Z*_1_ and *Z*_2_, and the intensity of the tail f relative to the intensity at the tube focus. In most instances the intensity of the tails is ≈0.1 % of the peak intensity and is only significant when analysing intense lines. The tails themselves are not necessarily symmetric with respect to the tube focus and can extend over a 2*θ* range up to 0.6°.

### 4.2 X-Ray Receiving System Models

In diverging beam diffractometers the receiving slit is placed at the focus of the diffracted beam and for perfect focussing should have an infinitely small width. Owing to the many aberrations present, focussing is never perfect and the count rate incident on the receiving slit tends to increase with increasing slit width, but at the expense of resolution. In parallel beam diffractometers the receiving system is based on using either the Hart-Parrish system of analyser slits [18] or, a flat analyser system as illustrated in Fig. 5 earlier. In many glancing incidence diffractometers the receiving system consists of analyser slits and an analyser crystal in the diffracted beam. Although the aberration functions associated with the various receiving systems for parallel beam and divergent beam diffractometers all possess different shapes, they all possess the common property of being independent of 2*θ*.

#### 4.2.1 Receiving Slit in a Diverging Beam Diffractometer

Most commercial diffractometers have a selection of receiving slits ranging in width from 0.05 mm up to 0.3 mm although occasionally larger slit sizes up to 0.6 mm are used to measure integrated intensity rapidly. The aberration function for a perfectly aligned receiving slit is an impulse function of width Δ2*θ*_r_ given by,

$$\Delta 2\theta_{r}=\frac{w_{r}}{R}\text{rad}$$ (5)

where *w*_r_ is the width of the receiving slit. The angular width Δ2*θ*_r_ subtended by the receiving slit relative to the diffractometer axis is therefore normally between 0.013° (0.05 mm) and 0.08° (0.3 mm). When the slit size is larger than 0.15 mm, the receiving slit aberration is often the dominant aberration in a diffractometer over the angular range 2*θ* = 15° to 60°.

#### 4.2.2 Parrish-Hart Analyser Slits

**Fig. 8:**
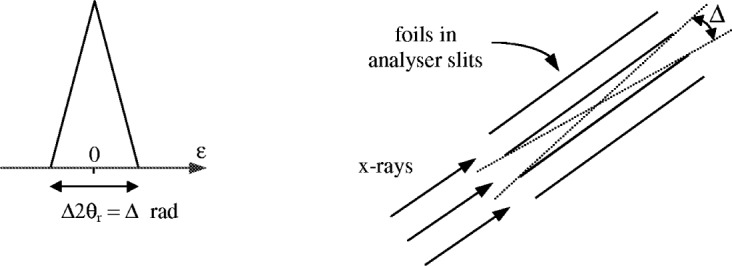
Triangle shaped aberration function for a set of analyser slits with an angular aperture *Δ* where *Δ*/2 = spacing between the foil/length of the foils.

Analyser slits act as an angular filter in the diffracted beam. The aberration function or transmission function for these slits is a triangle function, as shown in Fig. 8, in which the base width Δ2*θ*_r_ is given by the angular aperture *Δ* of the slits.

**Fig. 9:**
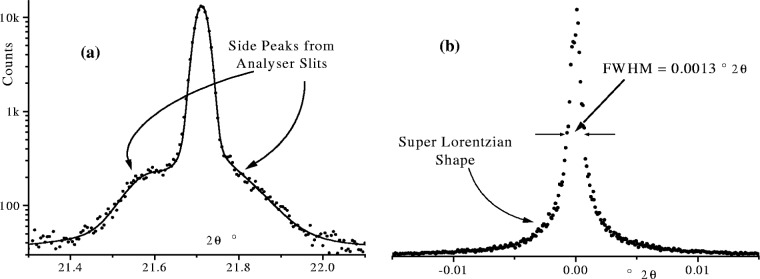
(a) Reflection satellite peaks from analyser slit recorded using the 310 line from NIST standard material LaB6 SRM 660a using the diffractometer on Station 2.3 at Daresbury synchrotron. (b) 2*θ* scan across a 0.1 mm × 0.1 mm incident beam using a Ge111 analyser crystal on beamline BM16 at the ESRF, Grenoble.

In the original Parrish-Hart diffractometer on Station 2.3 at the Daresbury synchrotron, the analyser slits were 360 mm with a spacing of 0.2 mm between adjacent foils giving an angular 2*θ* aperture *Δ* ≈ 0.06°. In laboratory diffractometers the angular aperture *Δ* is typically ≈0.1°. A problem often encountered with analyser slits is specular x-ray reflection from the analyser foils [19]. Weak satellite peaks appear on both the high angle and low angle profile tails but not necessarily of the same intensity as shown in Fig. 9a. This effect has been incorporated into the aberration profile by adding two Voigt functions of unequal intensity, one on each side of the triangular aberration function, to represent the satellite reflections [11]. The parameters of the satellite peaks can then be determined by fitting profiles from a reference material such as the NIST reference standard LaB_6_, SRM 660a. An alternative approach to determining the aberration profile of an analyser slit system, without the effects of the wavelength profile distorting the result, is to simply carry out a 2*θ* scan across the incident beam. Provided the axial divergence of the incident beam is kept small, by including axial Soller slits, and the equatorial divergence is negligible then the incident beam scan will have exactly the same shape as the aberration profile of the analyser slits.

#### 4.2.3 Analyser Crystals

The inclusion of an analyser crystal in the diffracted beam of a parallel beam diffractometer gives high resolution diffraction patterns with a low background, but the intensity is invariably less than the Parrish-Hart configuration. The aberration profile introduced by the analyser crystal is generally very narrow and can be determined by measuring the rocking curve of the crystal. For a perfect analyser crystal the aberration profile will be determined by the Darwin profile of the analyser crystal. In practice, however, the aberration profile will be broadened by the mosaic structure of the crystal, any stresses in the crystal and any waviness or curvature of the crystal surface [20]. As a consequence the aberration profile can be dependent on the size of the beam incident on the crystal. A first approximation to the shape of the aberration profile of an in-situ analyser can be obtained from a 2*θ* scan of the analyser/detector using a very fine incident beam as shown in Fig. 9b. Although the profile recorded in this way also has the wavelength distribution folded into it, the result does at least give an indication of the shape and upper limit of the FWHM of the aberration profile.

### 4.3 Flat Specimen Error

**Fig. 10:**
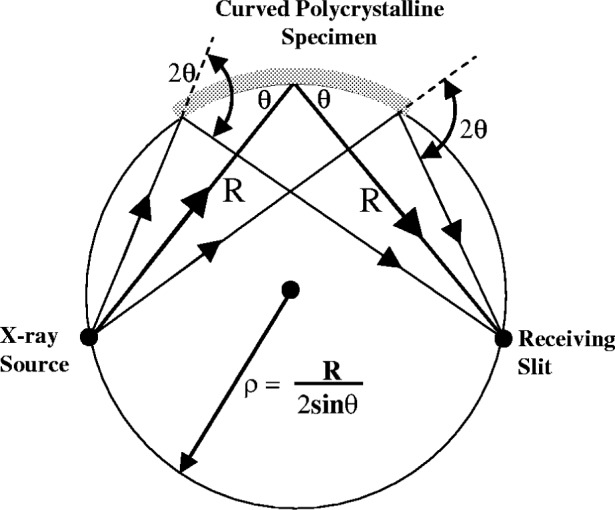
Focussing in a symmetric powder diffractometer.

The basic optics of the focussing powder diffractometer set up for symmetric diffraction is illustrated in Fig. 10. The x rays are incident at an angle *θ* on an ideal polycrystalline specimen with a surface radius of curvature *ρ*. For diffraction from a particular *hkl* plane the common property of all the diffracted rays from the specimen is that they all deviate through the same angle 2*θ*. By simple geometry it can be shown that all the diffracted rays converge to a focus on a circle which has the same curvature as the specimen surface. The focus of the diffracted rays defines the position of the receiving slit. In commercial diffractometers the specimen is invariably flat and the diffracted beam no longer focusses perfectly. Good focussing characteristics, however, can be maintained with appropriately chosen slits to limit the equatorial divergence and a diffractometer radius *R* sufficiently large to reduce defocussing errors to an acceptable level without losing too much diffracted intensity.

**Fig. 11:**
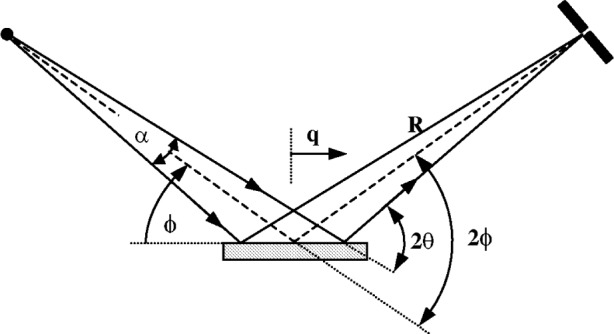
Diffraction from a flat plate showing the relationship between the measured angle 2*ϕ* on the diffractometer and the diffraction angle 2*θ* for a ray at the outer limit of a beam of divergence *α*.

For an incident beam, with an equatorial divergence *α* centred on the diffractometer axis, the aberration profile *J*_FS_(2*θ*) is asymmetric and exists only for the region *ε* = 0. X rays diffracted from the centre of the specimen are detected at 2*ϕ* = 2*θ* where as x rays diffracted off-centre are detected at 2*ϕ* < 2*θ* as shown in Fig. 11.

The relationship between the difference *ε* = 2*ϕ* − 2*θ* and the distance *q* from the diffractometer axis at which the ray is diffracted is,

$$\varepsilon =-{\left(\frac{q}{R}\right)}^{2}\sin2\theta \;\text{rad}$$ (6)

assuming small angles of divergence for the incident beam (eg. *α* < 2°). Thus, when a specimen is illuminated over a length *Q* then the x rays diffracted from either extremity of the beam (ie., at *q* = ±*Q*/2) defines the limiting value of *ε*_M_ of the aberration function *J*_FS_(*ε*) which exists within the range 0 ≥ *ε* ≥ *ε*_M_ where

$$\varepsilon_{M}=-{\left[\frac{Q}{2R}\right]}^{2}\sin2\theta \;\text{rad}$$ (7)

This relation can also be expressed in terms of the equatorial divergence of the beam *α* starting from the relation,

$$Q=\frac{\alpha R}{2}\left(\frac{1}{\sin(\theta -\alpha /2}+\frac{1}{\sin(\theta +\alpha /2}\right).$$ (8)

When 2*θ* > 10°, Eq. (8) can be approximated as

$$Q=\frac{\alpha R}{\sin\theta }$$ (8a)

and *ε*_M_ becomes

$$\varepsilon_{M}=\frac{\alpha^{2}}{2}\cot\theta \;\text{rad}.$$ (9)

When the incident beam is centred on the diffractometer axis the normalised equation for the aberration function *J*_FS_(*ε*) for flat specimen error is,

$$J_{\text{FS}}\left(\varepsilon \right)=\frac{1}{2\sqrt{\varepsilon \varepsilon_{M}}}\;\text{for}\;0\geq \varepsilon \geq \varepsilon_{M}.$$ (10)

**Fig. 12:**
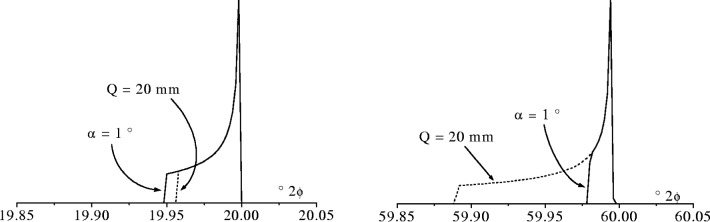
Comparison of flat specimen aberration functions at 2*θ* = 20° and 2*θ* =60° for a diffractometer with either a fixed divergence angle *α* = 1° or fixed illumination length *Q* = 20 mm (*R* = 215 mm). All the aberration functions shown were convoluted with a very narrow Lorentzian emission profile with a FWHM = 0.001 mÅ to overcome infinities at *ε* = 0. Each plot is normalised to the same *I*_max_ and both plots cover a range of 0.2° 2*θ*.

Modern commercial diffractometers operate with either a fixed divergence *α* or a fixed illumination length *Q*. In the fixed *α* mode the aberration function is broad at low 2*θ* and *ε*_M_ has a cot *θ* dependence whereas in fixed *Q* mode the breadth rises from zero at 2*θ* = 0 up to a maximum at 2*θ* = 90°. The extent of the changes in the aberration function *J*_SF_(*ε*) for each mode of operation using typical operating values for *α* and *Q* are shown in Fig. 12.

With a fixed angle of divergence *α* = 1°, the effects of flat specimen error in commercial diffractometers are discernable as an increase in both the asymmetry and breadth below 2*θ* ≈ 40° [21]. Under conditions of constant *α* the beam size increases with decreasing 2*θ* and eventually the beam will cover the whole specimen. The angle 2*θ*_lim_ at which this occurs is given by,

$$\sin\left(\theta_{\lim}\right)=\alpha R/L_{\text{sp}}$$ (11)

where *L*_sp_ is the length of the specimen. Under these circumstances the value of *ε*_M_ is given by Eq. (7) with *Q* = *L*_sp_. Below 2*θ*_lim_ the aberration function remains the same as the beam extends beyond the specimen.

In diffractometers with a fixed illuminated length, the effects of flat specimen error are generally smaller at low 2*θ* values and increase with increasing 2*θ*. With an illuminated length of 20 mm on the specimen, flat specimen error is clearly discernable at 2*θ* > 20°. It is not always possible to collect data from fixed *Q* mode diffractometer over a large 2*θ* range (eg., up to 150° 2*θ* as the *α* angle required at large 2*θ* is larger than the diffractometer can accommodate. For example, to maintain a fixed beam length of 20 mm on a specimen over the range 2*θ* = 0° to 90°, the angle of divergence *α* will need to increase from 0° up to ≈4°. A divergence angle *α* = 4° is close to the maximum value at which most diffractometers can operate when a pyrolytic graphite monochromator is installed in the diffracted beam. At angles of *α* greater than 4° the diffracted beam may extend beyond the graphite crystal and not be diffracted into the detector. In practice, this is not usually a problem as fixed *Q* mode diffractometry is normally used for analysing materials such as clays with very low angle diffraction lines where the lines of interest start at 2*θ* ≈ 3°.

### 4.4 Specimen Transparency

Specimen transparency produces asymmetry and broadening of the instrumental profile function. For perfect focussing all the diffraction should occur on the focussing circle, but when the beam penetrates the surface, diffraction will occur over a range of depths within the specimen. The aberration function *J*_µ_(*ε*) for an infinitely thick specimen is given by,

$$J_{\mu }\left(\varepsilon \right)=\frac{\exp\left(\varepsilon /\delta \right)}{\delta }\;\varepsilon \leq 0$$ (12)

where δ = (2/*µR*) sin2*θ* rad and *µ* is the linear attenuation coefficient. In low absorbing specimens which cannot be considered to be infinitely thick, the angular variable *ε* has a lower limit *ε*_min_ so that in Eq. (12),

$$0\geq \varepsilon \geq \varepsilon_{\min}\;\text{and}\;\varepsilon_{\min}=-\frac{2T}{R}\cos\;\theta \;\text{rad}$$ (12a)

where *T* is the specimen thickness. As a consequence the unit area normalising constant in (12) also changes and *J*_µ_(*ε*) becomes,

$$J_{\mu }\left(\varepsilon \right)=\frac{\exp\left(\varepsilon /\delta \right)}{\delta \left[1-\exp\left(\varepsilon_{\min}/\delta \right)\right]}.$$ (12b)

**Fig. 13:**
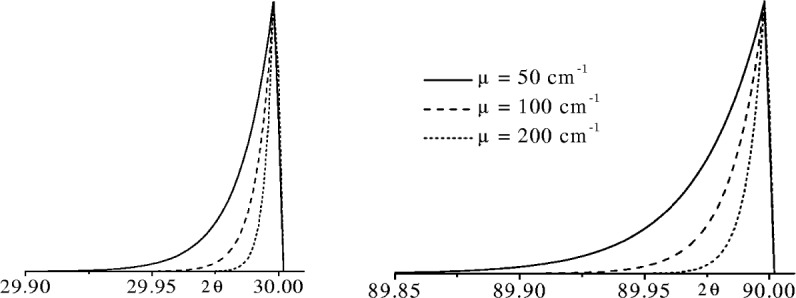
Specimen transparency aberration profiles at 2*θ* = 30° and 90° for linear attenuation coefficients of 50, 100, and 200 cm^−1^.

The asymmetry and broadening from specimen transparency is greatest for low absorption materials and is clearly evident when the linear attenuation coefficient *µ* < 50 cm^−1^. The contribution of specimen transparency is greatest at 2*θ* ≈ 90° and at this angle the aberration profile has a FWHM ≈ 0.03° 2*θ* when *µ* ≈ 50 cm^−1^, but this drops to ~0.005° 2*θ* when *µ* ≈ 200 cm^−1^. Fig. 13 shows the shapes of aberration profiles for an infinitely thick specimen and how they are affected by both *µ* and 2*θ*. Specimen transparency effects are quite strong in polymeric materials with very low attenuation coefficients (*µ* ≈ 30 cm^−1^ or less). They can also show up in loosely bound powders of low atomic number materials (e.g., MgO or Si) where the porosity ≈50 % or less so that a material with a *µ* = 100 cm^−1^ is reduced to a powder with a *µ* = 50 cm^−1^.

### 4.5 Diffractometer Defocussing

Defocussing results in broadened diffraction lines and occurs when the receiving slit is not positioned at the focus of the diffracted beam. The most common causes of defocussing are,

- mis-setting the incident beam angle *ω* so that it is no longer at the symmetric condition *ω* = *θ* as in asymmetric diffraction or,
- wrongly positioning the receiving slit so that the distance of the slit from the sample is larger or smaller than the nominal radius *R* of the diffractometer.

**Fig. 14:**
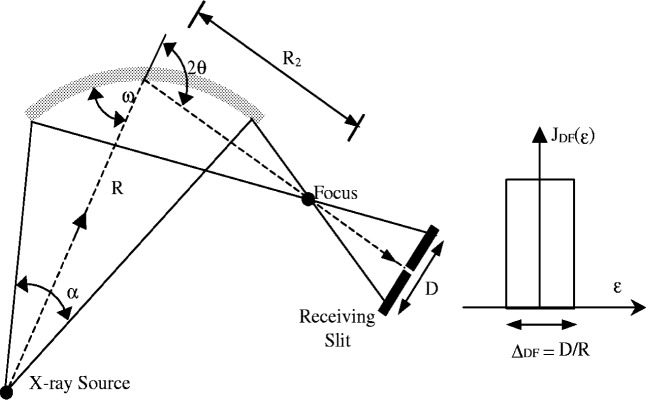
Defocussing under asymmetric diffraction conditions giving an impulse shaped aberration function *J*_DF_(*ε*) of angular width *Δ*_DF_.

For both of these conditions the focus of the diffracted beam will be either in front of or behind the receiving slit as illustrated in Fig. 14 for the asymmetric diffraction case.

The aberration profile *J*_DF_(*ε*) for this condition is a impulse function of angular width *Δ*_DF_ = *D*/*R* (rad) where *D* is the width of the defocussed beam at the receiving slit. Assuming the equatorial divergence *α* is small (ie., 2° or smaller) the angular width *Δ*_DF_ of the aberration profile is given by

$$\Delta_{\text{DF}}=\frac{D}{R}=\alpha \left|\frac{R_{2}-R}{R}\right|$$ (13)

Under asymmetric diffraction conditions *R*_2_ and *R* are related by the equation,

$$R_{2}=R\frac{\sin\left(2\theta -\omega \right)}{\sin\;\omega }$$ (14)

so that the width *Δ*_DF_ of the aberration function is,

$$\Delta_{\text{DF}}=\alpha \left|1-\frac{\sin\left(2\theta -\omega \right)}{\sin\;\omega }\right|.$$ (15)

**Fig. 15:**
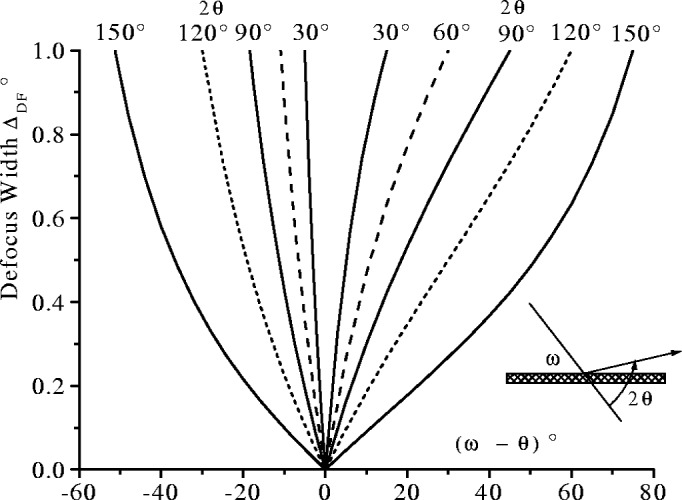
The variation of defocussing width *Δ*_DF_ with (*ω* − *θ*) at different 2*θ* diffraction angles.

In asymmetric diffraction, the breadth of diffraction lines increases as the deviation from the symmetric condition, |*ω* − *θ*|, increases. Defocussing is larger at low 2*θ* angles and varies more rapidly with *ω* − *θ* at low 2*θ* values. Conversely, at high 2*θ* values, diffractometers will tolerate quite large errors in *ω* − *θ* (ie., up to ±5° at 2*θ* ≈ 150°) without the effects of defocussing being detectable in the line breadth. Also, by reducing the angle of divergence *α* the defocussing *Δ*_DF_ can also be reduced, but at the expense of the diffracted intensity. The plot of *Δ*_DF_ vs (*ω* − *θ*) for a range of 2*θ* angles from 30° to 150° is shown in Fig. 15.

### 4.6 Axial Divergence

In a laboratory diffractometer only a small fraction of the photons that form the incident beam emerge from an x-ray source parallel to the equatorial plane. Likewise most of the x rays that reach the detector slit after diffracting from the sample are angled to the equatorial plane. Under these circumstances, a diffractometer will record x-ray counts over a range of angles 2*ϕ* other than the true diffraction angle 2*θ*. The only rays for which 2*ϕ* = 2*θ* will be those propagating parallel to the equatorial plane and incident on the diffractometer axis. In practice, axial divergence is most readily recognised by the asymmetry it introduces into low angle diffraction lines (2*θ* < 30°) where the low angle tails extend further than the high angles tails.

**Fig. 16:**
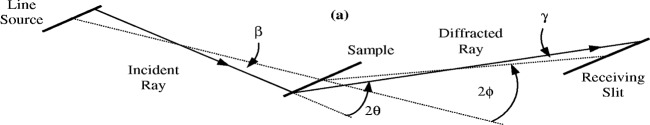
An illustration of axial divergence in which the incident ray and diffracted ray are at angles of *β* and *γ* relative to the equatorial plane. Although the diffraction angle at the sample is 2*θ*, the diffracted beam is recorded at an angle 2*ϕ.*

For a particular ray path the measured diffraction angle 2*ϕ* for a true diffraction angle 2*θ* depends on the axial divergence *β* and *γ* in the incident and diffracted rays (see Fig. 16). Assuming small angles for *β* and *γ*, the difference *ε* = 2*ϕ* − 2*θ* is given by,

$$\varepsilon =2\phi -2\theta =\beta \gamma \text{cosec}2\theta -\frac{\beta^{2}+\gamma^{2}}{2}\cot2\theta .$$ (16)

From this equation it is evident that the effect of axial divergence on *ε* will not only be large when 2*θ* is small, but also when 2*θ* is large (ie., 2*θ* > 150°) where the asymmetry is opposite to that at low 2*θ*. Although axial divergence is as strong in very high angle lines as it is in low angle lines, the positive asymmetry developed tends to be less noticeable as it is overshadowed by the dispersion of the emission profile.

When axial divergence in the incident beam is small (*β* ≈ 0) as in many synchrotron systems or laboratory diffractometers with very narrow incident beam Soller slits, all the axial divergence arises from the diffracted beam and Eq. (16) reduces to,

$$\varepsilon =-\frac{\gamma^{2}}{2}\cot2\theta .$$ (16a)

**Fig. 17:**
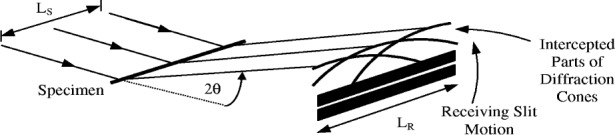
Arcs formed in the vicinity of the receiving slit by the diffraction cones radiating from all points across the sample axis. The axial divergence aberration function is the profile shape detected by an infinitely narrow receiving slit as it scans through the arcs.

For this condition, axial divergence is absent at 2*θ* = 90°, *ε* ≤ 0 when 2*θ* < 90° and *ε* ≥ 0 when 2*θ* > 90°. The aberration function *J*_AX_(*ε*) for this condition can be derived analytically [22,23,3,4] by considering an axially parallel beam from a line source incident on a narrow capillary specimen of randomly oriented crystallites as shown below in Fig. 17.

**Fig. 18:**
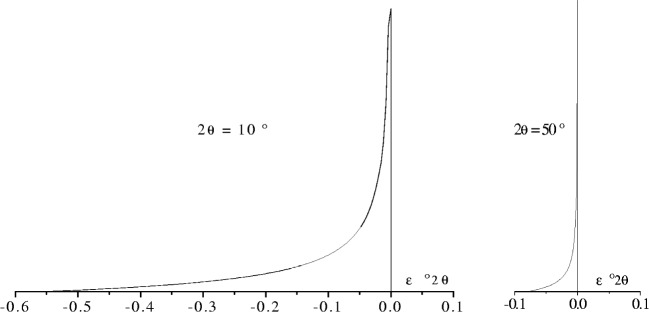
Axial divergence aberration profiles *J*_AX_(*ε*) for a diffractometer with no axial divergence in the incident beam. This was calculated using Eq. (17) for *L*_S_ = 10 mm, *L*_R_ = 15 mm, and *R* = 215 mm at 2*θ* = 10° and 2*θ* = 50°. A very narrow Lorentzian profile was convoluted with *J*_AX_(*ε*) to overcome the infinities at *ε* = 0.

The emergent diffracted beam from each individual ray in the incident beam in Fig. 18 is a collection of radiating cones with a semi-angle 2*θ* and *J*_AX_(*ε*) can be derived by determining the intensity profile when the receiving slit is scanned across these cones. The parameters of the diffractometer that define *J*_AX_(*ε*) in this instance are *L*_s_, the axial length of the specimen bathed in x rays and the length *L*_r_ of the receiving slit and the function *J*_AX_(*ε*) is given by,

$$J_{\text{AX}}\left(\varepsilon \right)=\frac{1}{\left|\varepsilon_{1}-\varepsilon_{2}\right|}\left(\sqrt{\frac{\varepsilon_{2}}{\varepsilon }}-\sqrt{\frac{\varepsilon_{1}}{\varepsilon }}\right)\;\text{for}\;\varepsilon =\left\{0,\varepsilon_{1}\right\}$$ (17a)

$$\begin{array}{l} J_{\text{AX}}\left(\varepsilon \right)=\frac{1}{\left|\varepsilon_{1}-\varepsilon_{2}\right|}\left(\sqrt{\frac{\varepsilon_{2}}{\varepsilon }}-1\right)\;\text{for}\;\varepsilon =\left\{\varepsilon_{1},\varepsilon_{2}\right\}\\ \text{where}\;\varepsilon_{1}=-\frac{\cot2\theta }{2}{\left(\frac{L_{r}-L_{s}}{2R}\right)}^{2}\;\text{rad}\\ \text{and}\;\varepsilon_{2}=-\frac{\cot2\theta }{2}{\left(\frac{L_{r}+L_{s}}{2R}\right)}^{2}\;\text{rad}. \end{array}$$ (17b)

Examples of this aberration function at 2*θ* = 10° and 50° are shown in Fig. 18 for conditions that resemble a laboratory diffractometer with very narrow incident beam Soller slits (ie., ≤1°), *R* = 215 mm, an illuminated specimen length *L*s = 12 mm and a receiving slit of length *L*r = 16 mm.

When allowance is made for axial divergence in both the incident and diffracted beams and for the presence of Soller slits in each of these beams, the calculation of the aberration function *J*_AX_(*ε*) can no longer be done analytically. Eastabrook [24] first demonstrated how to calculate the axial divergence aberration profile for a conventional diffractometer, but restricted his discussion to instrumental conditions that could be solved analytically rather than for conditions that were widely used in practice. Pike [25] generalised the application conditions and included the effect of Soller slits, but restricted his calculations to the determination of the centre of gravity and variance of profiles rather than the aberration profile itself. More recently Cheary and Coelho [3,4] have developed a semi-analytical approach to the calculation and their results have been incorporated into a profile refinement procedure. In short, their procedure consists of,

- calculating the analytical aberration function *J*_AX_(*β*,*ε*) arising from incident rays all with the same axial divergence *β*. The instrument parameters required to define this function are the axial lengths *L*_x_, *L*_s_, and *L*_r_ of the x-ray source, the sample and the receiving slit,
- incorporating the Soller slits into the calculation as angular intensity filters on the axial divergence *β* and *γ* in the incident and diffracted beams, respectively. The transmission functions, *S*_I_(*β*) and *S*_D_(*γ*), for the incident and diffracted beam Soller slits, respectively, are each triangle functions with 100 % transmission at *β* = 0 and *γ* = 0, and 0 % transmission at *β* = ± *Δ*_I_/2 and *γ* = ± *Δ*_D_/2 where *Δ*_I_ and *Δ*_D_ are the angular apertures of the slits (see Fig. 9 for definition of *Δ*),
- calculate the full aberration profile *J*_AX_(*ε*) by integrating the aberration functions determined at each *β* across all the allowed *β* values,
  $$J_{\text{AX}}\left(\varepsilon \right)=\int_{\beta_{\min}}^{\beta_{\max}}J_{\text{AX}}\left(\beta ,\varepsilon \right)S_{I}\left(\beta \right)S_{D}\left(\gamma \left[\beta ,\varepsilon \right]\right)d\beta .$$ (18)
  In practice this integration is converted to a summation, but great care is needed carrying out the summation because of singularities in the *J*_AX_(*β*,*ε*) functions. Considerable savings in computing time without loss of accuracy can be achieved by using a smoothing procedure in which the function *J*_AX_(*ε*) is convoluted in *ε* space with an impulse of width = step size of the data being fitted. The saving in computing time comes from a reduction in the number of summation points at discrete values of *β* required to accurately reproduce Eq. (18) using a summation.

In the absence of Soller slits the maximum axial divergence in the incident and diffracted beams, $\Delta_{I}^{\max}$, and $\Delta_{D}^{\max}$ respectively, is determined by the length *L*_x_, *L*_s_, and *L*_r_ and given by,

$$\tan\Delta_{I}^{\max}=\frac{L_{x}+L_{s}}{R}\;\text{and}\;\tan\;\Delta_{D}^{\max}=\frac{L_{s}+L_{r}}{R}.$$ (19)

**Fig. 19:**
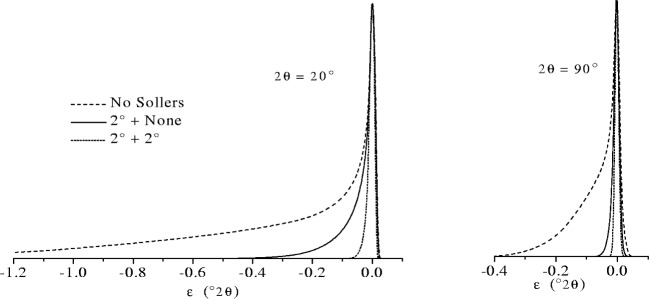
Axial divergence aberration profiles *J*_AX_(*ε*) calculated for various combinations of incident beam and diffracted beam Soller slits (or no Soller slits) 2*θ* = 20° and 2*θ* = 90°. Instrument conditions: Diffractometer radius 215 mm, *L*_X_ = 12 mm, *L*_S_ = 25 mm, *L*_R_ = 12 mm. The three combination used are (i) No Soller slits, (ii) 2° Soller slits in the incident beam only and, (iii) 2° Soller slits in both the incident and diffracted beams. All the aberration profiles are normalised to the same *I*_max_.

Under these circumstances the axial divergence can be as large as 10° in the incident beam and 10° in the diffracted beam for a diffractometer with a 12 mm long x-ray source, a 12 mm receiving slit and a 20 mm wide specimen. However, most commercial diffractometers are supplied with Soller slits having angular apertures *Δ* between 2° and 5°, and in such cases the breadth of the aberration profile is reduced quite dramatically. This is illustrated in Fig. 19 which shows aberration profile shapes calculated using the method of Cheary and Coelho for diffractometer configurations that include Soller slits as well as a configuration with no Soller slits. When narrow Soller slits are included in the beam path (ie., *Δ*_I_ and *Δ*_D_ ≈ 2°), the dimensions *L*_x_, *L*_r_, and *L*_s_ tend to be redundant parameters in the calculation of *J*_AX_(*ε*) as the Soller slits control the maximum axial divergence in the incident and diffracted beams.

The major change brought on by axial divergence is extension of the low angle tails of profiles below 2*θ* ≈ 50°. At very high angles, 2*θ* ≥ 150°, the asymmetry reverses and the extension of the high angle tails increases with increasing 2*θ*. Broadening from axial divergence is evident at all 2*θ* values but passes through a minimum in the region 2θ ≈ 110°. However, the shift in peak angle 2*θ*_max_ at *I*_max_ relative to the true 2*θ*_B_ is small at all 2*θ* values unlike the shift in centre of gravity 2*θ*_cg_ − 2*θ*_B_ which varies quite considerably from very large negative values, near 2*θ* = 0°, to very large positive values as 2*θ* approaches180° [12].

**Fig. 20:**
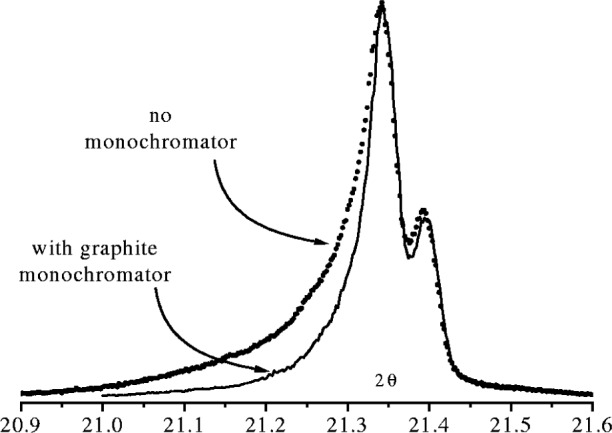
Effect on the 100 profile from LaB_6_ powder of including a graphite monochromator in the diffracted beam in a diffractometer with no diffracted beam Soller slits. The two profiles were normalised to the same peak intensity *I*_max_.

In practice it is not always possible to calculate the exact form of the axial divergence aberration function for a particular specimen/diffractometer configuration. The two main reasons for this are,

- in specimens with strong preferred orientation, such as thin films and rolled or extruded metals, the diffraction cones are no longer of uniform intensity along the arcs of the diffraction cones.
- the inclusion of a monochromator in the beam path reduces the axial divergence. When either diffracted beam or an incident beam monochromator is present the optical path length of the beam is extended. With a diffracted beam monochromator the optical path length of the diffracted beam is extended by the optical path between the receiving slit and the detector slit. In a diffractometer with a graphite monochromator tuned to Cu K*α* radiation the optical path length in the diffracted beam is increased by ≈100 mm giving an effective radius of the detector arm of 100 + *R* mm. An example of the extent to which axial divergence is reduced by introducing a graphite diffracted beam monochromator is illustrated in Fig. 20. Incident beam monochromators also increase the optical path length of the incident beam. For the most common type of monochromator in use, the asymmetrically Ge ground and bent monochromator, the path is increased by over 300 mm. Monochromators also act as angular intensity filters and their effect on a profile is similar to the addition of Soller slits in the beam path [26]. The effect of a monochromator can therefore be represented as a Soller slit in a profile fitting model.

### 4.7 Linear Position Sensitive Detector (LPSD) Aberrations

LPS detectors with angular windows up to 10° (at 200 mm) are used in reflection mode on commercial diffractometers to increase the data collection rate particularly for kinetic based studies. In most cases LPS detectors are used in stationary mode and only a fixed angular region of a pattern is recorded. A number of manufacturers offer LPSD systems where the detector can be scanned in *θ* −2*θ* mode with its centre maintained at the focus condition. As the detector is scanned the total diffraction pattern is formed by adding together and averaging the patterns recorded at each step. In this mode a diffraction pattern can be accumulated very rapidly with excellent counting statistics because individual lines are within the detector window for a considerable time. For example a LPSD with an angular window of 10° moving at 5° per min corresponds to a diffraction peak being detected for 120 s. Consequently, in this mode, a 100° 2*θ* diffraction pattern may only take approximately 20 min to collect. To obtain the same level of counting precision in a conventional single slit diffractometer with 0.02° 2*θ* steps would take approximately one week. In practice, this gain in time is never fully realised because of the longer dead times of LPSDs and the damage that can be caused on the anode wire by very high localised count rates. Also the diffraction peaks recorded at off-centre positions along the detector window are broadened and asymmetric. Some manufacturers make the angular window a software adjustable parameter to maintain the resolution although this means that only a fraction of the detector is being used.

In LPSD systems, the receiving slit aberration is no longer relevant and the flat specimen aberration is replaced by an aberration function that embodies three effects which are folded together in the final function;

- flat specimen error including defocussing,
- parallax error,
- thermal noise.

A full treatment of these aberrations and how they are modified by a scanning LPSD system is given in Cheary and Coelho [14]. For a stationary LPSD the aberrations are discussed in Secs. 4.7.1–4.7.3.

#### 4.7.1 LPSD Flat Specimen Error *J*_PSD_(*ε*) Including Defocussing

**Fig. 21:**
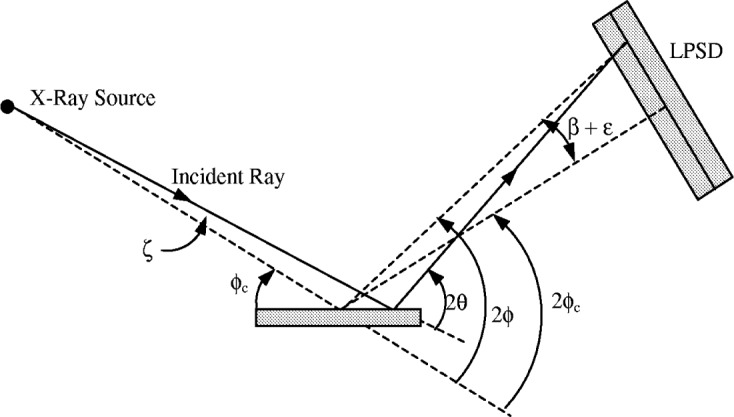
Beam geometry for an LPSD diffractometer showing the various angular parameters used in the derivation of the aberration function *J*_PSD_(*ε*).

In a conventional diffractometer defocussing and flat specimen error can be convoluted together independently, but this is no longer valid in a LPSD system. The effect of both the specimen and the detector being flat is that the defocussing is no longer symmetric about the centre of the LPSD. In a stationary LPSD the aberration function *J*_PSD_(*ε*) depends on the 2*θ* value of a peak and on the offset angle *β* of the recorded peak from the centre of the LPSD. The angular variables used to define the LPSD system are given in Fig. 21 where the *ζ* is limited by the angle of divergence *α* of the incident beam (ie., −*α*/2 ≤ *ζ* ≤ +*α*/2).

When the diffractometer is operated symmetrically and the incident beam is centred on the diffractometer axis then assuming small angles for *ε* and *ζ*, the difference *ε* = 2*ϕ* − 2*θ* is related parabolically to *ζ*,

$$\varepsilon =a\zeta -b\zeta^{2}\;\text{or}\;{\left(\zeta +\zeta_{0}\right)}^{2}=\frac{\varepsilon_{0}-\varepsilon }{b}\;\left({}^{\circ}2\theta \right)$$ (20)

where $a=\cos\beta \frac{\sin(\theta +\beta /2}{\sin(\theta -\beta /2}-1$, $b=\frac{\pi }{180}\frac{\sin2\theta \cos\beta }{\sin^{2}\left(\theta -\beta /2\right)}>0$, $\varepsilon_{0}=\frac{a^{2}}{4b}$. and $\zeta_{0}=\frac{a}{2b}$. The equation for the aberration function *J*_PSD_(*ε*) is obtained by transforming the intensity across the incident beam IB(*ζ*) from *ζ* space into *ε* space, ie.,

$$J_{\text{PSD}}\left(\varepsilon \right)=\text{IB}\left(\zeta \right)\left|\frac{d\zeta }{d\varepsilon }\right|$$ (21)

**Fig. 22:**
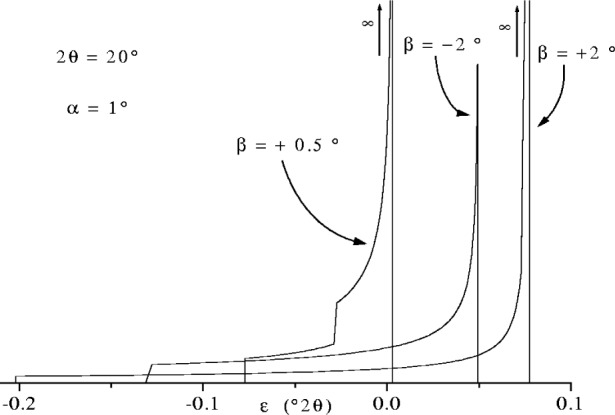
LPSD flat specimen error/defocussing aberration function *J*_PSD_(*ε*) for an incident beam of divergence *α* = 1° at 2*θ* = 20°. Examples are given of *J*_PSD_(*ε*) near the centre of the PSD (*β* = 0.5) and at 2° either side of centre (ie., *β* = +2° and −2°).

**Fig. 23:**
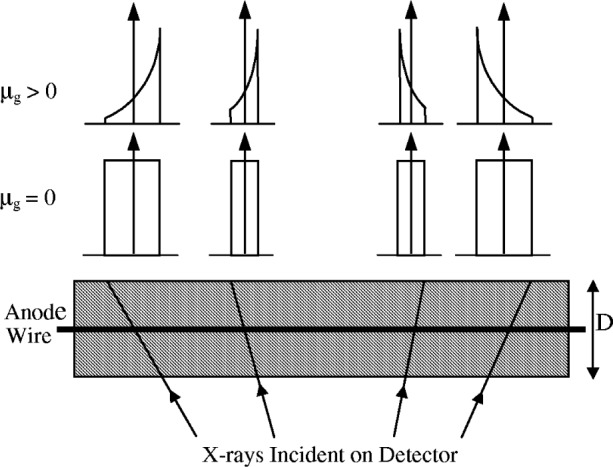
Aberration profiles for parallax error and the effect of the absorption *µ*_g_ of the detector gas.

As the incident beam intensity IB(*ζ*) is reasonably constant then the un-normalised form of *J*_PSD_(*ε*) = |d*ζ*/d*ε*|. This is readily calculated by differentiating the transformation Eq. (20) and has the form,

$$J_{\text{PSD}}\left(\varepsilon \right)=\frac{K}{\sqrt{\varepsilon_{0}-\varepsilon }}$$ (22)

where *ε* ≤ *ε*_0_. When calculating this function allowance has to be made for the fact that Eq. (20) can be double valued. Physically this means that more than one part of the incident beam contributes at a particular *ε* value and *J*_PSD_(*ε*) possesses a discontinuity at the boundary between two rays and one ray contributing to the aberration function. This is illustrated in Fig. 22 for profile arising from a central ray entering the LPSD at 0.5° off-centre when set at a low 2*θ* (ie., *β* = 0.5°, 2*θ* = 20°). When *ε*_0_ is outside the range of *ε* values dictated by the limiting values of *ζ* in the incident beam (typically ±0.5°) then there are no discontinuities within *J*_PSD_(*ε*), no infinities and *J*_PSD_(*ε*) is finite at all *ε* as illustrated in Fig. 23 for the profile with *β* = −2°. It should be noted that the aberration functions for a particular 2*θ* are not symmetric with respect to *β*. In Fig. 22 the profiles for *β* = +2° and *β* = −2° at 2*θ* = 20° are distinctly different and have different limits in *ε*.

#### 4.7.2 Parallax Error

**Fig. 24:**
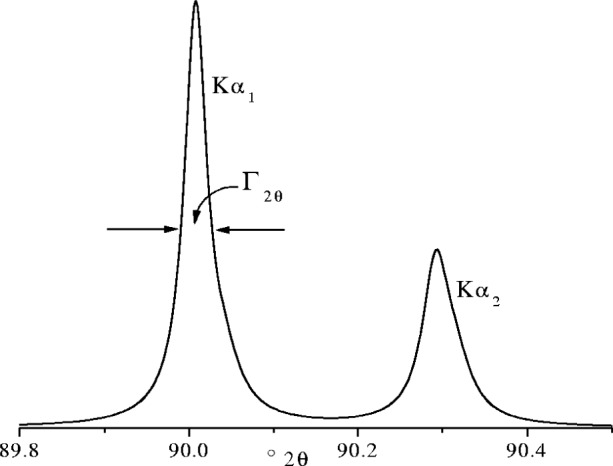
K*α*_1_*α*_2_ emission profile from copper recorded using the 400 line from a silicon single crystal.

When a diffracted x-ray photon enters a LPSD its path in the detector gas is not perpendicular to the anode wire, except at the centre of the detector, and additional broadening, known as parallax broadening, is introduced into off-centre diffraction lines. This arises because the ionisation caused by incoming photons is likely to occur at any point along its path, but the subsequent avalanche is perpendicular to the anode wire (see Fig. 24). For a detector gas with low absorption, the profile shape recorded by the LPSD at an angle ±*β* from the centre of the LPSD is a unit area impulse function with a width *Δ*_PX_ (°2*θ*) given by,

$$\Delta_{\text{PX}}=\frac{\left|\beta \right|D}{R}$$ (23)

where *D* is the depth of the detector and *R* is the radius of the diffractometer. Parallax error can be reduced considerably by increasing the detector gas pressure. In this way the x rays are absorbed by a thin layer of gas beneath the detector window, in which case the diffractometer radius *R* is defined by the front window of the detector rather than its anode wire.

Most detectors are designed with a quantum efficiency of at least 80 % and have depths somewhere between 5 mm and 10 mm. The aberration profiles for these conditions resemble those shown in Fig. 23 where the absorption of the detector *µ*_gas_ > 0. The shape of the aberration profiles for parallax broadening *J_µ_*_PSD_(*ε*) at an angle *β* to the centre of the LPSD is given by,

$$J_{\mu \text{PSD}}\left(\varepsilon \right)=\frac{\mu_{g}R}{\left|\beta \right|}\frac{\exp\left(-\frac{\mu_{g}\varepsilon R}{\beta }\right)}{\left[1-\exp\left(-\mu_{g}D\right)\right]}$$ (24)

where *µ*_g_ is the linear attenuation coefficient of the detector gas [27]. Parallax broadening can be quite large for long detectors and is usually the dominant aberration when LPSDs are used for peaks above 2*θ* ≈ 40°. For example, when *β* = ±5° and the detector has a depth *D* ≈ 8 mm, the breadth of the parallax function *Δ*_p_ can be as large as 0.2°.

#### 4.7.3 Thermal Noise

The ultimate resolution of a LPSD is the spatial uncertainty of the position measurement of an individual x-ray photon incident normal to the anode wire of the detector. This is controlled by the spatial broadening of the discharge produced by incoming photons, by the thermal noise generated in the anode wire and by the accuracy of the position-encoding electronics [28]. In most instances the thermal noise profile *J*_NPSD_(*ε*) is modelled as a Gaussian function with a FWHM *σ*,

$$\text{TN}\left(\varepsilon \right)=\left(2/\sigma \right)(\text{ln}{\left(2\pi \right)}^{1/2}\text{exp}\left[-\text{ln}\left(2\right)\left(2\varepsilon /\sigma \right)2\right]$$ (25)

This function represents the distribution of “measured positions” of a discharge caused by an incoming x-ray photon when the photon is incident normally at exactly the same position along the wire. In most LPSDs, the angular width *σ* is equivalent to a positional uncertainty along the anode wire between *Δx* ≈ 0.04 mm and 0.20 mm where *σ* = *Δx*/*R* radians. This effect contributes to the broadening much like that from the typical receiving slit width in a conventional diffractometer. Smaller *Δx* values down to 0.040 mm are possible when the gas in the LPSD is under pressure. Most LPSDs have a positional resolution *Δx* around 0.1 mm although those that utilise delay lines can be as large 0.2 mm.

## 5. The Wavelength Distribution in Laboratory Diffractometers

The natural shape of the energy distribution *W*(*E*) of a single characteristic x-ray emission line well above the threshold energy is Lorentzian and given by

$$W\left(E\right)=\frac{\Gamma /2\pi }{{\left(E-E_{0}\right)}^{2}+{\left(\Gamma /2\right)}^{2}}$$ (26)

where *E*_0_ is the peak emission energy and *Γ* is the lifetime broadening given by the sum of the widths of the two relevant atomic levels involved in the transition.

When a Lorentzian energy spectrum is transformed into 2*θ* space it remains Lorentzian, provided d*E*/d2*θ* is relatively constant across the profile, with a FWHM *Γ*_2_*_θ_* in 2*θ* space, given by

$$\Gamma_{2\theta }\approx \frac{2\Gamma \tan\theta }{E_{0}}\frac{180}{\pi }\;\left({}^{\circ}2\theta \right)$$ (27)

where *Γ* and *E*_0_ are in eV. The form of the Kα_1_α_2_ emission profile from Cu is shown in Fig. 24. For all of the transition element anodes used in x-ray diffraction neither the Kα_1_ line nor the Kα_2_ line has a Lorentzian shape and both the Kα_1_ and Kα_2_ lines are asymmetric with extended high angle tails. Moreover, the asymmetries and FWHM values of the Kα_1_ and Kα_2_ peaks are different. This is particularly evident with the first series of transition element and is related to the anomaly in the atomic number dependence of the atomic level widths of the L_II_ and L_III_ levels obtained from x-ray photoelectron spectroscopy data and x-ray emission spectroscopy [29]. The line widths *Γ*, asymmetry indices *κ* and energies *E*_0_ of the four transition elements commonly used for x-ray targets are given in Table 1.

**Fig. 25:**
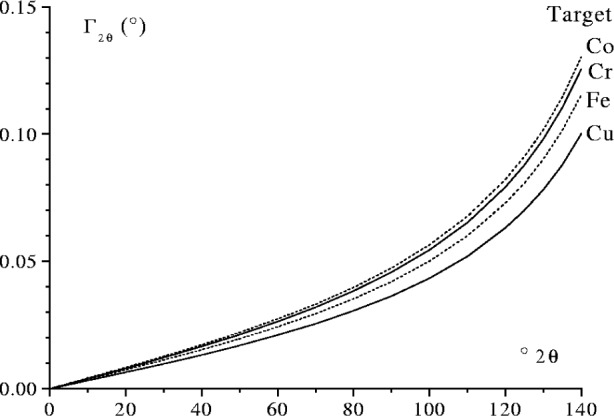
Full width at half maximum intensity of the Kα_1_ line *Γ*_2_*_θ_* as a function of 2*θ* for four different target materials.

In FPPF an accurate model of the emission profile is essential particularly for the analysis of high angle profiles. The FWHM *Γ*_2_*_θ_* of the Kα_1_ line from Cu and other commonly used transition metal anodes is shown in Fig. 25. At 2*θ* = 40° the FWHM *Γ*_2_*_θ_* is less than or equal to 0.01° (2*θ*) is considerably smaller than the FWHM values of actual diffraction lines from commercial diffractometers which are typically in the range 0.07° to 0.10° 2*θ* [21] depending on the choice of slits. In this region the contribution of the emission profile is swamped by the geometrical aberrations. When 2*θ* > 60°, however, the emission profile tends to dominate over the geometrical aberrations. Once 2*θ* > 100°, the total breadth of the geometrical aberrations is relatively minor and the profile shape conforms closely to the emission profile.

**Fig. 26:**
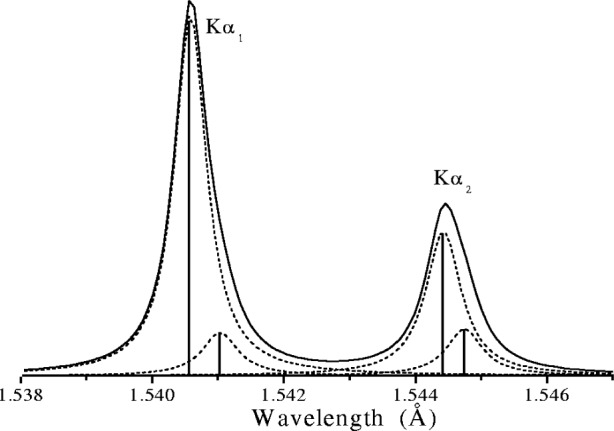
Phenomenological representation of the Cu Kα emission profile based on four Lorentzians.

The natural asymmetry of the emission lines arises from the multiplet structure of the transitions. In addition to the main transitions involving the change in vacancy state 1*s* → 2*p*, it has been recognised that 3*d* spectator transitions also contribute up to 30 % of the Kα_1_α_2_ emissions [31]. In these transitions the atom is doubly ionised and the actual vacancy transition is still 1*s* → 2*p*, but the second vacancy in the 3*d* level is not directly involved in the transition. The notation for this transition is 1*s*3*d* → 2*p*3*d*. A phenomenological representation for accurately describing the asymmetric Cu emission profile was first used by Berger [32]. In this model the Kα_1_ and Kα_2_ lines were each represented as two Lorentzian profiles as shown in Fig. 26. This representation was also used successfully on accurate spectroscopic data from Cu obtained by Härtwig et al. [33]. A systematic study of the Kα and Kβ emission profiles from Cr, Mn, Fe, Co, Ni, and Cu [34] has shown that the phenomenological representation can be used to accurately represent these elements down to an *R* factor of 1 %, although in some cases it is necessary to use up to seven Lorentzians.

**Fig. 27:**
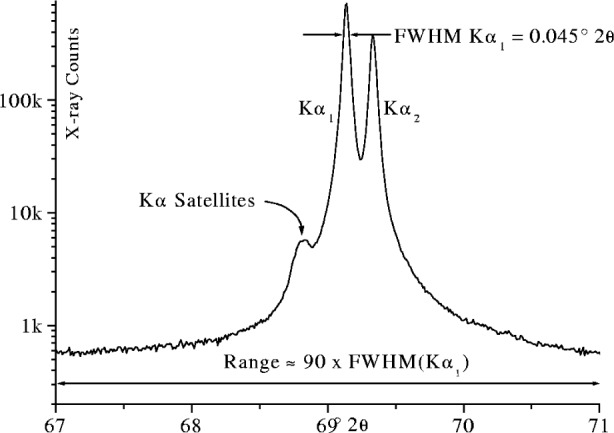
Cu Kα emission profile showing the satellite group of lines and the extent of the tails from the Kα1 and Kα2 emission lines. This profile was recorded using the 400 line from a silicon single crystal wafer.

Another feature of Kα emission lines which needs to be included in an accurate line profile fitting model is the satellite multiplet structure in the high energy tail as shown in Fig. 27. As a group these have an intensity of ≈0.6 % of the Kα_1_ emission line in the case of Cu rising uniformly with decreasing atomic number up to ≈1.4 % for Cr [35]. Satellites lines are also evident in Kβ_1_β_3_ spectra and appear on both the low energy and high energy tails as the Kβ′ and Kβ″ lines [36]. The Kα_3_α_4_ non-diagram lines arise from the transition 1*s*2*p* → 2*p*^2^ in which the actual vacancy transition is 1*s* → 2*p*, but in the presence of a 2*p* spectator hole [37,38]. For most of the first transition series the Kα satellite structure can be fitted accurately with four or five Lorentzians [35,38]. In most x-ray diffraction studies this level of precision in fitting the satellites is unnecessary. For Cu it is sufficient to represent the Kα satellite group as a single broad Lorentzian so that the total Kα spectrum can be represented by five Lorentzians as given in Table 2.

In the transformation of the distribution *W*(*λ*) from wavelength space to 2*θ* space at 2*θ* < 130° the Lorentzian shape in *λ* maps directly into a Lorentzian in 2*θ* with minimal error. At higher angles the effects of dispersion between *λ* and 2*θ* become increasingly evident [12] and the transformation needs to be carried more accurately. The full expression for the transformed wavelength distribution in 2*θ* space for a sum of Lorentzians in *λ* space is

$$\begin{array}{c} W\left(2\theta \right)=W\left(\lambda \right)\frac{d2\theta }{d\lambda }\\ =\frac{1}{d\;\cos\;\theta }\sum_{i=1}^{5}\frac{I_{0i}\left(\Gamma_{\lambda i}/2\pi \right)}{\left[{\left(\Gamma_{\lambda i}/2\right)}^{2}+{\left(2d\sin\theta -\lambda_{i}\right)}^{2}\right]} \end{array}$$ (28)

where *I*_0_*_i_* and *Γλ_i_* are the relative areas and wavelength FWHM of the *i*th Lorentzian. The *d* spacing is defined in relation to the reference wavelength *λ*_ref_ of the emission line which in the TOPAS implementation of this procedure is the wavelength of the highest intensity Lorentzian. In the case of the Cu Kα spectrum given in Table 2, the reference wavelength is that of the Kα_1a_ component (*λ* = 1.540591 Å) so that *λ*_ref_ = *λ*_Kα1a_ = 2*d* sin*θ*_Kα1a_. At very high 2*θ* values (ie., 2*θ* ≥ 150°) the cos*θ* terms arising from d2*θ*/d*λ* can change quite significantly over a profile and elevate the intensity in the high angle tail. In addition to dispersion, the distortional effects of the Lorentz factor on high profiles should also be incorporated when analysing profile shapes at very high angles [39,40,41].

**Fig. 28:**
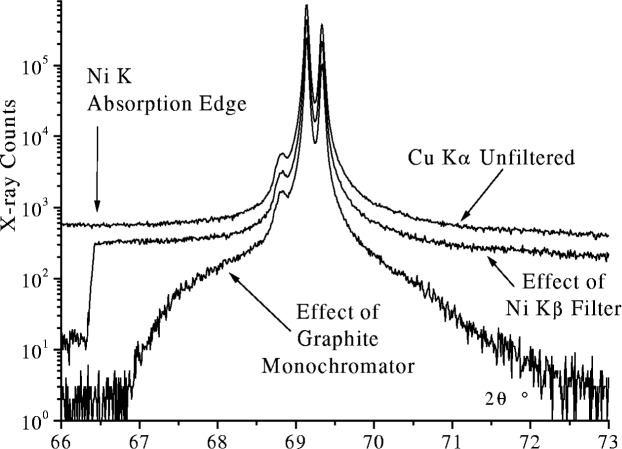
Cu Kα emission profile obtained using the 400 line from a silicon single crystal wafer. Each pattern was recorded sequentially using the same sample, first with no filter or monochromator in the beamline, then with a NiKβ filter, and finally with a standard curved graphite diffracted beam monochromator.

As the natural shape of an emission profile is Lorentzian, the tails can extend a considerable distance from the central peak as shown in Fig. 27. Most diffractometers, however, operate with Kβ filtering or some form of monochromatisation and as such the tails are attenuated to varying degrees. When a Ni Kβ is included in the beamline of Cu Kα instrument, the attenuation appears to be more or less uniform across the profile except below the Ni K absorption edge. Careful analysis however, reveals a small variation in the attenuation across the profile owing to the increase in linear attenuation coefficient with increasing wavelength (ie., µ α λ^3^), but this is generally a small effect and is not expected to affect the profile shape significantly [12]. The inclusion of a curved graphite monochromator in the diffracted beam of a diffractometer greatly reduces the range of wavelengths entering the detector resulting in profile tails that diminish more rapidly than the natural emission profile as shown in Fig. 28. These monochromators can also affect the relative intensity *I*_Kα2_/*I*_Kα1_ ratio by up to ±10 % depending on the alignment and setting of the crystal. For example, in the monochromated spectrum shown in Fig. 28 the relative intensity of the CuKα_1_/Kα_2_ is approximately 0.46 rather than 0.50 as in the unfiltered spectrum.

**Fig. 29:**
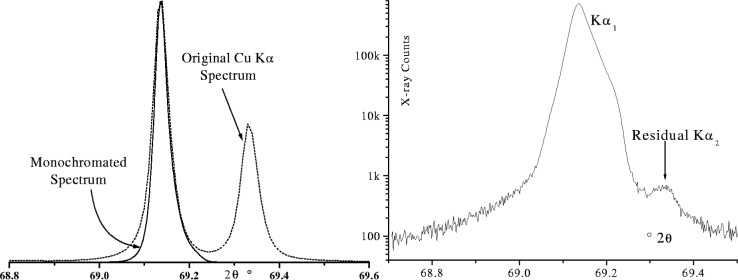
Wavelength spectrum emerging from an asymmetrically cut Ge111 ground and bent incident beam monochromator presented linearly and logarithmically. The Kα satellites are completely removed, but the Kα_2_ is still present at ≈0.02 % of Kα_1_ even in a well aligned system.

Commercial pyrolytic graphite monochromators are strongly oriented polycrystals bent by a hot pressing operation. The focussing is imperfect and the resolution is relatively poor. Better resolution and, in some cases, high intensities can be obtained from “ground and bent” (Johannson) single crystals. These crystals achieve perfect focussing when correctly aligned and are able to select a very narrow wavelength band. The most common materials used for Johannson monochromators on conventional diffractometers are quartz, germanium and silicon. The example shown in Fig. 29 shows the wavelength spectrum from an asymmetrically cut ground and bent Ge crystal used as an incident beam monochromator. The wavelength passband is narrow enough to remove 99.98 % of the Kα_2_ component and 100 % Kα satellites from the CuKα spectrum and, almost completely eradicate the Lorentzian tails of the emission profile.

In the presence of monochromators, even low resolution graphite monochromators, it is no longer possible to accurately model the wavelength distribution from an x-ray tube using the tabulated unfiltered spectra such as the one in Table 2. Although first principles calculations of the wavelength transmission function through ideal monochromator are possible, it is currently more practical to determine experimentally a “learned” spectrum for the curved graphite, germanium, quartz and lithium fluoride monochromators used in the majority of monochromated laboratory powder diffractometers. This can be done by modifying the “sum of Lorentzians” representation in energy space or *λ* space to fit the spectrum entering the detector. In broad terms, monochromators reduce the width of the wavelength distribution and tend to truncate the tails of spectra. A number of approaches can be used to accommodate these changes,

- represent the components of the wavelength distribution as Voigt or pseudo-Voigt functions rather than Lorentzians, to limit the extension of the profile tails, and modify the relative intensities of the component.
- represent the effect of the monochromator as a wavelength filter with a transmission function *T*(*λ*) represented by a simple function, such as a split pseudo-Voigt or split Pearson VII function with up to four refineable parameters, which operates on the tabulated Lorentzian emission profile data. The split functions are used to incorporate asymmetry in the *T*(*λ*).

In both cases the parameters of each representation are obtained by analysing and fitting the high angle profiles from either a reference line profile standard, such as LaB_6_ SRM 660a, or single crystal disc such as a 111 wafer of silicon.

**Fig. 30:**
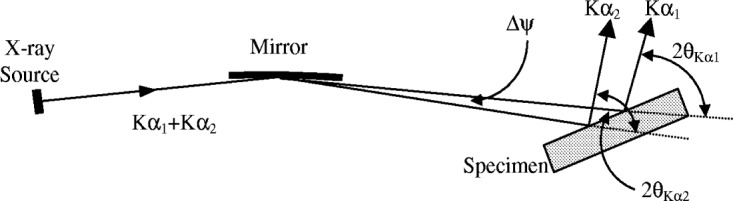
Reflections of Kα_1_ and Kα_2_ wavelengths from a parabolic multilayer mirror diffracting of a powder specimen.

**Fig. 31:**
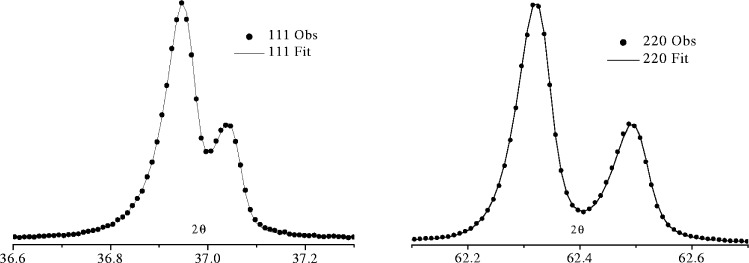
Results of fitting 111 and 220 profiles from MgO with the parameters shown in Table 3.

The inclusion of a parabolic multilayer mirror in the incident beam of a diffractometer can also introduce a distortion into the wavelength spectrum [42]. This happens because the Kα_1_ and Kα_2_ components of the spectrum reflect off the mirror in slightly different directions as shown in Fig. 31. The separation of the Kα_1_ and Kα_2_ peak maxima, *Δ*2*θ*_Kα21_ = 2*θ* (Kα_2_) − 2*θ* (Kα_1_), in a line profile is then either larger or smaller than the same profile from a diffractometer with no mirror. When a diffractometer is set up with its mirror in the orientation shown in Fig. 30, the separation *Δ*2*θ*_Kα21_ is smaller than the value expected from the known Kα_1_ and Kα_2_ wavelengths by an amount corresponding to the difference Δ*ψ* in the directions of the two incident beams on to the specimen. According to Toraya and Hibina [42] the difference *δψ* for a conventional long fine focus x-ray with a projected target width of 0.04 mm can rise to 0.0017° for a high resolution mirror, but decreases to a negligible level for low resolution mirrors. In any high accuracy lattice parameter determination using this type of diffractometer it is necessary to incorporate the wavelength dependent zero error into the analysis.

## 6. Fundamental Parameters Profile Fitting (FPPF) in Practice

In practice FPPF requires accurate numerical procedures for carrying out multiple convolution integrals. This can be done by representing the calculated profiles as a histogram and reducing the convolution integral to a summation. To avoid systematic errors with this approach the angular step size between calculated intensities needs to be very small but in so doing the operation becomes very time consuming [2]. In the XFIT and TOPAS implementations of FPPF a semi-analytical procedure has been developed for convoluting the aberration functions. In this procedure the two aberration functions being folded together are calculated at the same 2*θ* values as the measured data points and then a continuous function is formed by interpolating between the calculated points. As the calculated functions are then each a series of linear sections it is possible to calculate the convolution integral analytically [2]. Some difficulties are experienced with aberration functions that possess singularities, but in all cases the functions are integrable and the effect of the singularity can be overcome either by a convolution process or by a smoothing operation [4].

Although FPPF is a powerful method of profile analysis it can fail when used without recognising its limitations. The physical parameters describing a diffractometer are not statistically independent within the least squares refinement procedure and strong correlations exist between many of the refineable parameters. For example, when the angular acceptance angles, *Δ*_I_ and *Δ*_D_, of the incident beam and diffracted beam Soller slits are refined independently to a set of profiles, various combinations of refined values *Δ*_I_ and *Δ*_D_ can be obtained that give equally good fits to the data. The reason for this is that these two parameters are very strongly correlated and the values can in fact be interchanged without changing either the shape of a synthesised profile significantly or the quality of the fit. As a consequence the best fit with an unconstrained refinement often corresponds to refined parameters that do not make physical sense. When first using the FPPF approach it is advisable to investigate the validity of the refined parameters for a particular diffractometer using a well crystallised reference specimen and establish the parameters of the instrument that allow profiles over the whole 2*θ* range to be defined with one set of values. It would be unrealistic to expect the refined parameters for a diffractometer to match the directly measured values exactly as there are a number of second order effects in diffractometer profiles that are not incorporated in the fitting model. Moreover, not all of the instrumental aberrations are independent and as such the convolution model is not strictly valid with certain combinations of aberrations [8]. Nevertheless, experience with most of the commercially available diffractometers has shown that refined values reasonably close to the true instrumental values can be expected. In the original investigation of fundamental parameters fitting [2] it was shown that when deliberate changes were made to the diffractometer set-up, such as changing the receiving slit length, receiving slit width and diffractometer radii, the change in the refined instrument values corresponded well with the actual changes despite the limitations in the axial divergence model used at that time. In general, therefore, if physically unrealistic instrumental parameters are required to describe the diffraction pattern of a reference material then there is either a deficiency in the model used to describe the diffractometer, some sort of mis-setting of the diffractometer or the refinement is trapped in a false minimum.

Once the refined instrument parameters have been established (ie., the dimensions and apertures of the various slits, the source size and the wavelength distribution), and these are in reasonable agreement with the actual values, the only instrument parameter that may need refinement from specimen to specimen is the linear attenuation coefficient *µ*. Absorption effects in a line profile are only evident when *µ* < 100 cm^−1^ and under these circumstances there is some justification in making *µ* a refineable parameter. In specimens with *µ* ≥ 200 cm^−1^ refinement of *µ* normally has little effect on the profile shape because the specimen transparency profile is relatively narrow at all 2*θ* angles (ie., ≤0.01°2*θ*). Consequently, *µ* is fixed at a representative value and not refined.

Even in well crystallised powders with crystallite sizes up to 2 µm crystallite size broadening is detectable in high angle lines. Conversely, in many powders it is not uncommon to have crystallite sizes down to 0.5 µm and crystallite size broadening is evident even in low angle lines. Also, in many crushed powders the action of crushing even in a standard pestle and mortar can induce microstrain broadening. In TOPAS and XFIT therefore, the apparent crystallite size *T*_app_ and the percent microstrain *ε*_rms_ are frequently included as refineable parameters although their contribution is generally small. Various profile functions can be adopted for crystallite size and microstrain and commonly take the form of a Lorentzian function or a Gaussian function. The most common unit area functions used for crystallite size and microstrain, *B*_cryst_(2*θ*) and *B*_µ_(2*θ*), respectively, are

$$B_{\text{cryst}}\left(2\theta \right)=\frac{H_{\text{cryst}}/2\pi }{{\left(2\theta -2\theta_{0}\right)}^{2}+{\left(H_{\text{cryst}}/2\right)}^{2}}$$ (29a)

$$B_{\mu }\left(2\theta \right)=\frac{2}{H_{\mu }}\sqrt{\frac{\ln2}{\pi }}\exp\left[-\ln2{\left(\frac{2\left(2\theta -2\theta_{0}\right)}{H_{\mu }}\right)}^{2}\right]$$ (29b)

where the FWHM of the Lorentzian = *H*_cryst_ = 180 *λ*/(*πT*_app_ cos*θ*_o_) °2*θ* and the FWHM of the Gaussian = $H_{\mu }=\left(18\varepsilon_{\text{rms}}\sqrt{2\ln2}/5\pi \right)$ tan *θ*_0_ °2*θ*. In some crushed powders it has been necessary to use a Lorentzian function to represent the microstrain but with a FWHM = *η*tan*θ* where *η* is a constant related to the microstrain [4].

The quality of the fits obtainable from the FPPF approach and the degree to which the refined instrument parameters agree with the actual values is illustrated below for data collected from a polycrystalline MgO reference specimen over the range 2*θ* = 36° to 150° using CuKα radiation. The specimen used in this instance was a 20 mm disc prepared by sintering at 1400 °C for 24 h in air. When a small segment of the disc was examined in a scanning electron microscope the vast majority of crystallites were between 1 µm and 2 µm in diameter. Table 3 shows the refined values obtained for the MgO data from TOPAS whilst Fig. 30 shows the fits obtained to two of the profiles that are sensitive to the instrument parameters and the specimen absorption. Only four profile shape parameters were refined and these were the absorption coefficient *µ*, the receiving slit width *w*_r_, the angular aperture of the incident beam Soller slits *Δ*_I_ and the apparent crystallite size *T*_app_. All the other instrument parameters were fixed at their actual values. Some parameters, such as the equatorial divergence *α*, the width of the x-ray source and the sample width *L*_s_, have very little effect on the profile shapes and a wide range of values can be accommodated for each of these parameters without affecting the quality of the fit. Although the receiving slit length *L*_r_ and x-ray source length *L*_x_ can have a significant effect on the profile shape, they were not refined because of their strong correlation with the Soller slit aperture *Δ*_I_ and the fact that the improvement in fit with their inclusion was minimal. Equally good fits are obtained by fixing *L*_x_ and *Δ*_I_ and allowing *L*_r_ to refine. The equatorial divergence angle *α* and other axial divergence parameters are normally included in a refinement when low angle diffraction lines, preferably below 2*θ* = 25°, exist in the data set as this region of the diffraction pattern that is sensitive to equatorial and axial divergence. The inclusion of crystallite size broadening in most refinements is very important. In the present MgO refinement its removal from the refinement resulted in the other refined parameters taking on physically unrealistic values and a rise in the *R*_wp_ value from 3.5 % to 6.2 %. It was also clearly evident that the calculated profiles no longer fitted the tails of the observed profiles.

## 7. Concluding Remarks

Fundamental parameters profile fitting offers a number of benefits as a method of profile analysis. It is based on a physical model of the diffractometer and its refined parameters should be self consistent with physical dimensions of the diffractometer and the physical properties of the sample. On this basis it can therefore identify whether or not a diffractometer is operating at optimum resolution for the conditions used and provide a means for assessing the performance of a diffractometer in a particular application. For example, a knowledge of the performance of a diffractometer operating under asymmetric conditions can be important in choosing the best 2*θ* range and slits that will maintain sufficient resolution to monitor line positions in stress analysis. As the profile shape is known, the FPPF technique also provides greater certainty in the identification of weak peaks or impurity lines embedded in the tails of stronger line. In Rietveld analysis or quantitative analysis, FPPF allows the profile shapes across the whole 2*θ* range to be fitted without any instrument based parameters in the refinement. The focus of the refinement is therefore on the diffraction effects of the specimen and not the instrument. Moreover, line broadening analysis is an integral feature of FPPF for both individual lines or as part of a Rietveld analysis and, correction for instrument broadening is an intrinsic part of the analysis making reference specimens unnecessary in many circumstances. FPPF has its weaknesses. Up until now it has only had limited success as a method of accurate lattice parameter determination and in some diffractometer set-ups the FPPF instrument parameters can differ significantly from the actual values. Although it corrects the 2*θ* positions of lines for instrument line shift, including zero shift and specimen displacement, it has not been possible to obtain a set of lattice parameters for a particular specimen which are the same, within an uncertainty < ±0.0005 Å, for every *hkl* line in the pattern [43]. In addition, the FPPF approach does not have a physically based model for incorporating mirrors and monochromators into wavelength distribution and the axial divergence profile although recent work by Masson et al. [44] suggested an analytical approach in which devices within the optical path of a diffractometer can be readily incorporated into the theoretical formalism for describing the profile shape. The current physical modelling of laboratory based diffractometers and some of the diffraction processes within FPPF is adequate for many applications, but some degree of refinement of the theoretical procedures underpinning the technique is still necessary before the technique can claim to accurately describe both the shape and position of powder diffraction lines.

## Figures and Tables

**Table 1:** Line widths *Γ*, asymmetry indices *κ* and emission energies *E*_0_ for the Kα_1_ and Kα_2_ emissions of selected transition elements (data from Salem and Lee [30])

Element	*Γ*(eV)	Kα_1_ κ	*E*_0_(keV)	*Γ*(eV)	Kα_2_ κ	*E*_0_(keV)
Cr	2.16	1.38	5.415	2.75	1.18	5.406
Fe	2.35	1.43	6.404	2.84	1.25	6.391
Co	2.87	1.32	6.930	3.59	1.25	6.915
Cu	2.56	1.12	8.048	4.05	1.10	8.028

**Table 2:** Relative intensities *I*_0_ (areas), wavelengths *λ* and lifetimes widths *Γλ* (in *λ* units) for representing the Cu Kα spectrum by five Lorentzians. The Kα_1_α_2_ data were taken from Höltzer et al. [34] and the satellite data from Cheary and Coelho [2].

Emission line	*λ*(Å)	Relative *I*_0_	*Γ_λ_* × 10^3^ Å
Kα_1a_	1.540591	0.5710	0.437
Kα_1b_	1.541064	0.0789	0.643
Kα_2a_	1.544399	0.2328	0.513
Kα_2b_	1.544686	0.1036	0.687
Kα_3_α_4_ Satellites	1.534753	0.0137	3.686

**Table 3:** Comparison of actual diffractometer and sample parameters with values obtained for MgO data by FPPF refinement. In this refinement the radius of the diffractometer was fixed at 200 mm and the CuKα spectrum adopted for fitting was the one given earlier in Table 2. Note: NR denotes not refined

Physical variable	Actual value	Refined value
X-ray source width *w*_x_ (mm)	0.04	0.04 (NR)
X-ray source length *L*_x_ (mm)	12	12 (NR)
Incident Soller slits *Δ*_I_ (°)	2	1.85
Equatorial divergence α (°)	0.9	0.9 (NR)
Axial sample length *L*_s_ (mm)	20	20 (NR)
Absorption *µ* (cm^−1^)	74	68
Width of receiving slit *w*_r_ (mm)	0.13	0.095
Length of receiving slit *L*_r_ (mm)	10	10 (NR)
	Overall Fit R_wp_	3.5 %
	Expected R_wp_	2.4 %

## Footnotes

2 Certain commercial equipment, instruments, or materials are identified in this paper to foster understanding. Such identification does not imply recommendation or endorsement by the National Institute of Standards and Technology, nor does it imply that the materials or equipment identified are necessarily the best available for the purpose.
